# A Probability-Based Algorithm Using Image Sensors to Track the LED in a Vehicle Visible Light Communication System

**DOI:** 10.3390/s17020347

**Published:** 2017-02-10

**Authors:** Phat Huynh, Trong-Hop Do, Myungsik Yoo

**Affiliations:** School of Electronic Engineering, Soongsil University, Seoul 06978, Korea; phathuynhvn@gmail.com (P.H.); dotronghop@gmail.com (T.-H.D.)

**Keywords:** visible light communication, vehicle, tracking, probability, light-emitting diode, camera

## Abstract

This paper proposes a probability-based algorithm to track the LED in vehicle visible light communication systems using a camera. In this system, the transmitters are the vehicles’ front and rear LED lights. The receivers are high speed cameras that take a series of images of the LEDs. The data embedded in the light is extracted by first detecting the position of the LEDs in these images. Traditionally, LEDs are detected according to pixel intensity. However, when the vehicle is moving, motion blur occurs in the LED images, making it difficult to detect the LEDs. Particularly at high speeds, some frames are blurred at a high degree, which makes it impossible to detect the LED as well as extract the information embedded in these frames. The proposed algorithm relies not only on the pixel intensity, but also on the optical flow of the LEDs and on statistical information obtained from previous frames. Based on this information, the conditional probability that a pixel belongs to a LED is calculated. Then, the position of LED is determined based on this probability. To verify the suitability of the proposed algorithm, simulations are conducted by considering the incidents that can happen in a real-world situation, including a change in the position of the LEDs at each frame, as well as motion blur due to the vehicle speed.

## 1. Introduction

Vehicle visible light communication (V2LC) [1,2] is a promising application of visible light communication (VLC) because vehicles are equipped with high-power front and rear LED lights that can be utilized to transmit data. However, there are very few real-world applications of V2LC due to existing challenges in receiving the LED light signal. The receiver in a VLC system usually consists of a photodiode (PD) or an image sensor. However, a PD is well-known to be sensitive to ambient light, and thus is not suitable for use in outdoor applications, such as V2LC [3,4]. Image sensors can handle ambient light well (even sunlight), but problems remain when using image sensors for V2LC. For example, the frame rate of the camera is usually far less than what is needed in terms of the required data rate. This problem can somehow be solved as more and more high-speed cameras are introduced into the market at affordable prices. However, even when a high-speed camera is used to capture the series of LED images, it is not a simple task to track the position of the LEDs in each image because this must be done quickly and accurately. In addition, there are also difficulties related to the vehicle movement.

Different methods for using image processing for vehicle detection are reviewed in [5], and these detection algorithms are primarily used for detecting a whole vehicle. Therefore, they take into account many vehicle features, including color, symmetry structure, shadow, corner, edges, and various kinds of textures. These techniques are obviously more complicated than what is necessary for our simpler problem: to detect LED light of a vehicle. Furthermore, in the applications considered in this paper, there are likely many frames that need to be processed every second, and these techniques might not be fast enough.

In traditional methods [6,7,8,9], LEDs are tracked according to their intensity in the image. However, when the vehicles are moving, their images suffer from the motion blur effect, which makes it difficult to detect the LEDs using pixel intensity only. More specifically, when the LED image is blurred due to the motion of vehicles, all pixels belonging to the LED and background around the LED are blurred together and have about the same pixel intensity. Since there are more background pixels than that of LED pixels, the background pixels would be easily misdetermined as the LED pixels. Thus, when the speed is high, a missing frame can occur. As an example, an algorithm to track an LED array in a driving situation was proposed in [8]. This algorithm analyzes the pattern of the LED array. However, the mechanism to detect a single LED on the LED array still only relies on the pixel intensity in every frame.

This paper proposes a probability-based model to track the position of an LED in a series of images. The proposed algorithm uses not only the pixel intensity, but also the optical flow of an LED created in consecutive frames as well as statistical information from previous frames in which the LED was still clearly visible in order to calculate the probability that a pixel belongs to an LED or the background. Then, LED pixels in the image are determined based on this probability. When the LED is blurred due to vehicle motion, the LED pixels and nearby background pixels might have the same pixel intensity. However, the optical flow of the LED can still be constructed. The statistical information from previous frames are also taken into account to calculate the probability, and, thus, the LED pixels still have higher calculated probability than that of nearby background LED. In other words, the LED can be determined correctly even though it is blurred. The proposed algorithm is evaluated by conducting simulations considering incidents that happen in real-world scenarios, including change of the LED position in each frame and, in particular, the motion blur effect caused by the movement of the vehicle. The results show that our proposed algorithm significantly reduces the problem of missing frames when the vehicle is moving at high speeds.

## 2. Fundamental Background

### 2.1. System Architecture

**Figure 1:**
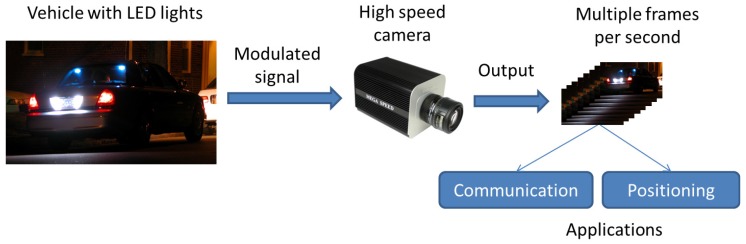
System architecture.

This paper considers a system where LED vehicle lights transmit visible light signals that will be received by a high-speed camera. The output of the camera consists of a series of images containing the LED. The data embedded in these images are then extracted for communication or positioning purposes. The whole system architecture is described in Figure 1, and this paper deals with the fundamental process of extracting the data encoded in the LED in the images.

### 2.2. Difficulty in Tracking the LED Light of a Moving Vehicle

**Figure 2:**
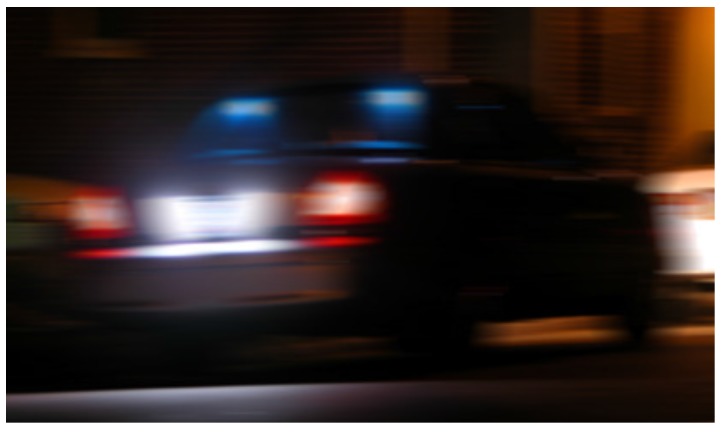
Motion blur effect.

One faces many difficulties in tracking the position of the LED light of a moving vehicle in a given set of images. The position of the LED can be simply found according to the pixel intensity, assuming that the pixels corresponding to the LED have the highest intensity. However, this method will fail to find the position of the LED in some scenarios. For example, when the vehicle is moving quickly, the LED image suffers from motion blur as illustrated in Figure 2. In this case, the LED image is blurred, and detecting the LED is much more difficult. In some cases, the LED in the image is hidden by obstacles in the street, which makes it impossible even to detect the LED.

## 3. Proposed Tracking Algorithm

Different types of information can be used to detect an LED in the image, and the conventional method mentioned above, for example, uses the pixel intensity. However, there are also other types of information that are useful to detect the LED, such as the optical flow of an LED and information from the previous frames where the LED was clearly visible. As a proper framework is required to use all these sources of information at the same time, this paper proposes a probability-based framework that takes all these information in consideration to determine the location of the LED in an image.

### 3.1. Information of a Pixel

#### 3.1.1. Optical Flow

The optical flow is one of the most important features in our model since the vehicle movement can be reflected in the optical signal captured by the image sensor. The optical flow is the pattern of apparent motion of the objects, surfaces, and edges in a visual scene created by the relative motion between the camera and the scene. Two consecutive images can thus be used to construct an instant optical flow map that shows the direction of the movement. The vehicle movement can then be measured on the images by taking two factors into consideration, the direction of the movement and the magnitude of the movement.

**Figure 3:**
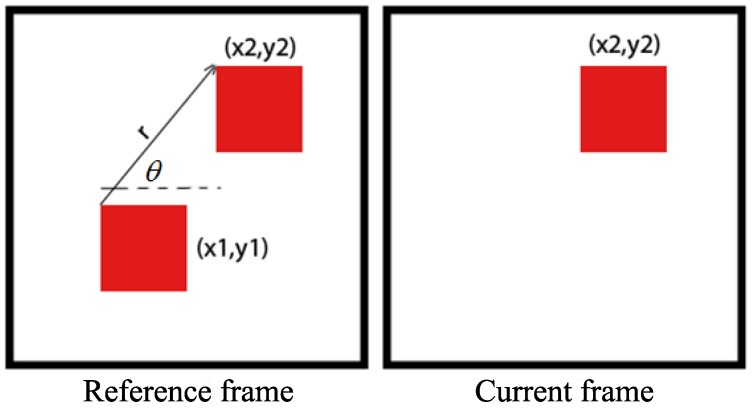
Motion vector construction.

The time interval between two consecutive frames is assumed to be very short, and thus the pixel intensities in two frames of the same LED are almost the same. Figure 3 illustrates how the motion vector of a pixel is constructed. The location of the pixel in the current frame is compared to the corresponding pixel in the reference frame in order to calculate the direction and magnitude of movement of that pixel. The direction of movement is then represented by the angle *θ*, while the magnitude of movement is denoted by *r*.

After calculating the motion vector for all pixels in the image, an effective motion vector map that is supposed to contain the pixels belonging to the vehicle is defined. The pixels belonging to the background are assumed to be stable or to just slightly change between consecutive frames. Then, the effective motion map is created by simply comparing the combination of *r* and *θ* to a given threshold. If the combination does not exceed the threshold, this pixel is considered to be stable. The following equation shows how to calculate the effective motion vector for every single pixel

(1) $${\mathrm{emv}}_{i}^{n}=\left\{\begin{array}{c} \begin{array}{cc} \delta (r_{i}^{n},\theta_{i}^{n}), & if\;\;\;\delta \left(r_{i}^{n},\theta_{i}^{n}\right)\geq \delta_{th}, \end{array}\\ \begin{array}{cc} 0,\;\;\;\;\;\;\;\;\;\;\;\;\;\;\;\;\;\;\;\;\;\;\;\;if & \delta \left(r_{i}^{n},\theta_{i}^{n}\right)<\delta_{th}, \end{array} \end{array}\right.$$

where *δ* is a metric from $R^{2}\mapsto R$, sub-index *i* stands for the $i^{th}$ pixel in the image, and upper-index *n* indicates the $n^{th}$ image in the series.

**Figure 4:**
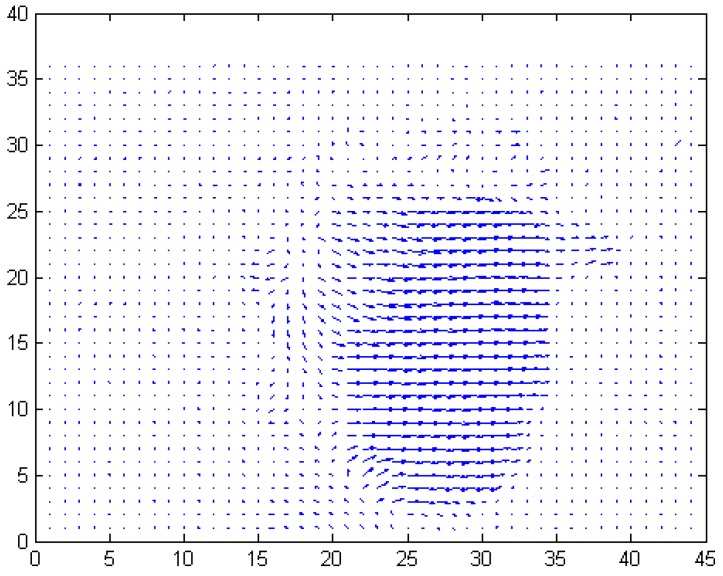
Effective motion vector map.

Figure 4 shows an effective motion vector map created from two consecutive frames. In this figure, the dots indicate the background pixels, and the arrows indicate pixels belonging to the vehicle.

#### 3.1.2. Pixel Intensity

The pixel intensity is the most basic feature to distinguish background pixels from LED pixels. In traditional tracking methods, the pixel intensity is compared to a threshold to determine if that pixel is an LED pixel or a background pixel. In this paper, the pixel intensity is used as one of the parameters to calculate the conditional probability that the pixel is an LED pixel.

Assume that the intensity of the *i*-th pixel of frame *n* is denoted as $f_{i}^{n}$. In normal image capturing, $f_{i}^{n}$ is assumed to have a Gaussian distribution over the image sensor, and this means that the pixel intensity would be the highest at the center, gradually decreasing toward the outer edge.

### 3.2. Probability-Based Tracking Algorithm

The optical flow and pixel intensity distribution are separate features of the image. To use them to find the position of the LED, we need to put them together into a single framework that takes both features as inputs. In this paper, the proposed Bayesian framework takes the optical flow and pixel intensity distribution as inputs to calculate the probability that a pixel belongs to the background or the LED.

**Figure 5:**
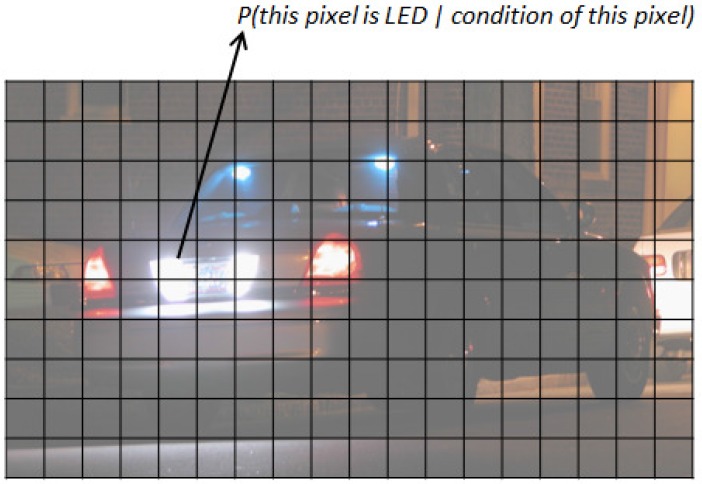
Concept of a probability-based tracking algorithm.

The basic idea of the proposed tracking algorithm is described in Figure 5. For every pixel in the image, the probability that the pixel belongs to an LED, given the known optical flow and intensity, is calculated. This is a conditional probability that can be calculated using Bayes’ rule.

First, a complete set of events is defined to determine where the pixel belongs. There are two states of a pixel: $\omega_{1}$ indicates that the pixel belongs to an LED, and $\omega_{2}$ indicates that the pixel does not belong to an LED. The probability function P is assumed to map the complete set of events to [0,1]. Let $inf_{i}^{n}$ be the information at the *i*-th pixel on frame *n*. This information contains two features mentioned above: the optical flow, which includes the direction and magnitude of the movement θ,r and the pixel intensity distribution *f*. At frame *n*, the conditional probability that the event $\omega_{1}$ or $\omega_{2}$ will occur is computed given the information from the series of previous images up to frame *n* given by Bayes’ rule:

(2) $$P_{i}^{n}\left(\omega_{1}|inf_{i}^{n}\right)=\frac{P_{i}^{n}\left(inf_{i}^{n}|\omega_{1}\right)P_{i}^{n}\left(\omega_{1}\right)}{\sum_{j=1}^{2}P_{i}^{n}\left(inf_{i}^{n}|\omega_{j}\right)P_{i}^{n}\left(\omega_{j}\right)},$$

where $inf_{i}^{n}=\left(emv_{n}^{i},f_{i}^{n}\right)$.

To calculate $P_{i}^{n}\left(\omega_{1}|inf_{i}^{n}\right)$, the two kinds of probability $P_{i}^{n}\left(inf_{i}^{n}|\omega_{j}\right)$ and $P_{i}^{n}\left(\omega_{j}\right)$ need to be calculated. Assume that *θ*, *r*, and *f* are statistically independent, the probability $P_{i}^{n}\left(inf_{i}^{n}|\omega_{j}\right)$ is given as:

(3) $$\begin{array}{cc} P_{i}^{n}\left(inf_{i}^{n}|\omega_{j}\right) & =P_{i}^{n}\left(r_{i}^{n},\theta_{i}^{n},f_{i}^{n}|\omega_{j}\right)\\ & =P_{i}^{n}\left(r_{i}^{n}|\omega_{j}\right)P_{i}^{n}\left(\theta_{i}^{n}|\omega_{j}\right)P_{i}^{n}\left(f_{i}^{n}|\omega_{j}\right), \end{array}$$

where j=1,2.

Assume that *θ*, *r*, and *f* have a normal distribution. The three likelihoods in Equation (3) are given by Gaussian functions as:

(4) $$\begin{array}{c} P_{i}^{n}\left(r_{i}^{n}|\omega_{j}\right)=\frac{1}{\sigma_{r,j}\sqrt{2\pi }}e^{-\frac{1}{2\sigma_{r,j}^{2}}{\left(r_{i,n}-\mu_{r,j}\right)}^{2}},\\ P_{i}^{n}\left(\theta_{i}^{n}|\omega_{j}\right)=\frac{1}{\sigma_{\theta ,j}\sqrt{2\pi }}e^{-\frac{1}{2\sigma_{\theta ,j}^{2}}{\left(\theta_{i,n}-\mu_{\theta ,j}\right)}^{2}},\\ P_{i}^{n}\left(f_{i}^{n}|\omega_{j}\right)=\frac{1}{\sigma_{f,j}\sqrt{2\pi }}e^{-\frac{1}{2\sigma_{f,j}^{2}}{\left(f_{i,n}-\mu_{f,j}\right)}^{2}}, \end{array}$$

where $\mu_{r,j},\mu_{\theta ,j},\mu_{f,j},\sigma_{r,j},\sigma_{\theta ,j},\sigma_{f,j},j=1,2$ are the mean and variance of the direction, amplitude of the optical flow and the pixel intensity calculated and updated throughout the previous frames.

Assume that the set of pixels that belong to the LED in the (n−1) frame is denoted by $ledPixels^{n-1}$. Then, the probability that a pixel belongs to an LED $P_{i}^{n}\left(\omega_{1}\right)$ is given by:

(5) $$P_{i}^{n}\left(\omega_{1}\right)=\frac{|ledPixels^{n-1}|}{\mathrm{total}\;\mathrm{number}\;\mathrm{of}\;\mathrm{pixels}},$$

where the $|ledPixels^{n-1}|$ denotes the number of LED pixels in the previous frame.

The probability that a pixel belongs to the background is simply given by:

(6) $$P_{i}^{n}\left(\omega_{2}\right)=1-P_{i}^{n}\left(\omega_{1}\right).$$

Then, the set of LED pixels in the current frame is defined as:

(7) $$ledPixels^{n}=\left\{i\in IS:P_{i}^{n}\left(\omega_{1}|inf_{i}^{n}\right)\geq P_{i}^{n}\left(\omega_{2}|inf_{i}^{n}\right)\right\}.$$

Equation (7) can be written as:

(8) $$ledPixels^{n}=\left\{i\in IS:P_{i}^{n}\left(inf_{i}^{n}|\omega_{1}\right)P_{i}^{n}\left(\omega_{1}\right)\geq P_{i}^{n}\left(inf_{i}^{n}|\omega_{2}\right)P_{i}^{n}\left(\omega_{2}\right)\right\}.$$

The decision rule in Equation (3) is applied over all pixels on the image to find the areas classified as LED areas. The exact location of the LED is determined by choosing the pixel with the highest probability of belonging to an LED:

(9) $$LED_{n}=\underset{i\in ledPixels}{arc\max}P_{i}^{n}\left(\omega_{i}|inf_{i}^{n}\right).$$

The advantage of the proposed algorithm can be seen through Equation (3). When the LED image is blurred due to the vehicle motion, pixels belonging to the LED and those belonging to nearby background are mixed together. Therefore, the pixel intensity now becomes less a decisive factor for tracking the position of the LED. This is the problem of the traditional tracking algorithms. However, in the proposed method, the probability on which the LED tracking is based is calculated from three probabilities as in Equation (3). The probability $P_{i}^{n}\left(f_{i}^{n}|\omega_{j}\right)$ of the LED pixels and nearby background pixels would have close values when the LED is blurred. However, the other two probabilities $P_{i}^{n}\left(f_{i}^{n}|r_{j}\right)$ and $P_{i}^{n}\left(f_{i}^{n}|\theta_{j}\right)$ still have higher value with LED pixels. Therefore, the LED still can be detected when it is blurred.

## 4. Simulation

### 4.1. Simulation Procedure

**Figure 6:**
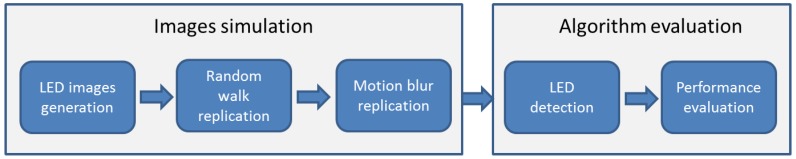
Simulation procedure.

The entire simulation procedure includes two steps, as described in Figure 6. First, a series of LED images is simulated using Matlab (R2011a, The MathWorks, Inc., Natick, MA, USA). Then, the traditional and proposed tracking algorithms are applied to these images and their performance is measured.

In the image simulation step, only one LED is assumed to be in each image. In addition, the frame rate of the camera is assumed to be 1000 fps. Thus, 1000 frames will be generated for every one second. In these frames, the LED images are clear and will have the same position. However, in a real-world situation, the position of the LED changes throughout the image series. A random walk model is applied to the simulated images to replicate the changes in the position of the LED, and a motion blur model is also applied to replicate the motion blur effect caused by the movement of the vehicle. After applying the two models, we obtain a series of frames where the position of LED changes slightly in each frame. Furthermore, the image of the LED can be blurred in some frames if the assumed vehicle speed corresponding to that frame is high.

In the second step, the simulated LED images obtained from the previous step are analyzed using a conventional method and the proposed tracking algorithms one after the other. The successful detection rates are then calculated to evaluate the performance of these algorithms.

#### 4.1.1. Random Walk Model

A modified random walk model is used to replicate the changes in the LED position in consecutive frames. The random walk normally includes two states, and in this paper, one more state is added to the model. Specifically, the direction of the vehicle is assumed to only change according to three states in a very short period of time: move up and move down with a constant degree, and move straight forward. A uniform distribution will be employed to trigger the direction in which the vehicle goes next. The following equation shows the moving direction of the vehicle:

(10) $$I_{t+1}=1_{\left\{U_{t+1}\leq \delta_{th}^{1}\right\}}I_{t}^{d}+1_{\left\{U_{t+1}\geq \delta_{th}^{2}\right\}}I_{t}^{u}+1_{\left\{\delta_{th}^{1}<U_{t+1}<\delta_{th}^{2}\right\}}I_{t}^{s},$$

where $I_{t+1}$ stands for the captured image at time t+1, U∼Uniform(0,1), upper indexes d,u,b stand for the down state, up state and stable state, respectively, and $\delta_{th}^{1},\delta_{th}^{2}$ are the chosen thresholds by which the direction could be determined—$\delta_{th}^{1},\delta_{th}^{2}\in \left[0,1\right]$ and $\delta_{th}^{1}<\delta_{th}^{2}$.

#### 4.1.2. Motion Blur Model

**Figure 7:**
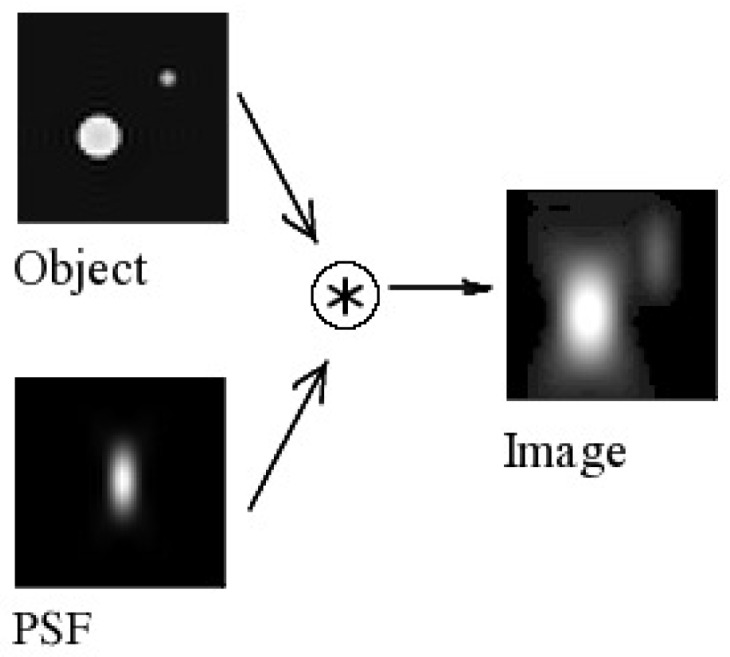
Object image blurred using the point spread function.

The blurring effect is one of the obstacles for LED tracking. In the literature, the blurring effect is described using a point spread function (PDF), applied to a clear image of an object to create a blurred image of that object. The PDF is described in Figure 7.

In this paper, a PSF model is adopted from Ref. [10]. This model takes the velocity and acceleration measured in pixels/second of the moving object as input, and the output is the number of pixels to which the object’s image is spread. More specifically, the PSF can be expressed in the case of constant speed as follows:

(11) $$p\left(x\right)=\frac{S\left(\frac{x}{v_{c}}\right)}{v_{c}},$$

and in the case of constant acceleration as follows:

(12) $$p\left(x\right)=\frac{S\left(\frac{-v_{s}+\sqrt{v_{s}^{2}+2a\times x}}{a}\right)}{\sqrt{v_{s}^{2}+2a\times x}},$$

where S(t) is the function of a binary shutter sequence, $v_{c}$ is the constant velocity, $v_{s}$ is the starting velocity, and *a* stands for the acceleration.

**Figure 8:**
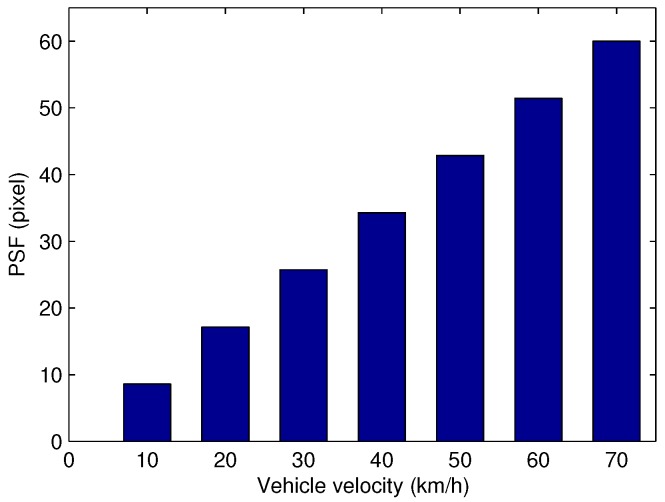
Point spread function over the vehicle speeds.

Regarding the simulation, the relationship between the point spread function and the vehicle speeds is described in Figure 8.

#### 4.1.3. Final Simulated Image

**Figure 9:**
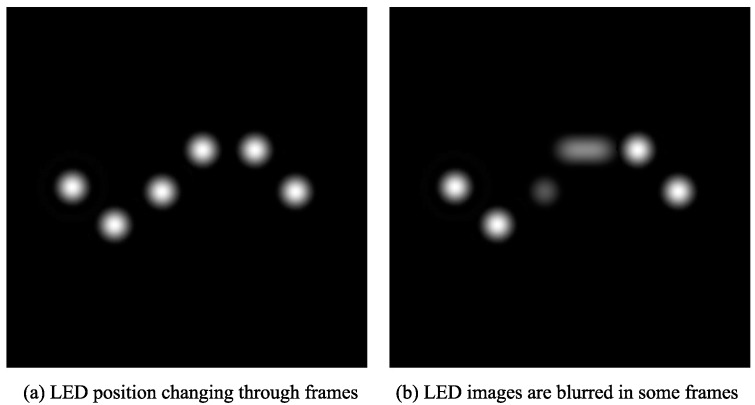
Image after random walk and motion blur model are applied.

The random walk model is applied starting from the initial simulated LED image to create images that are assumed to be the next frames. In these images, the position of the LED changes slightly, as shown in Figure 9a. Note that there is only one LED in each simulated image. In Figure 9, multiple frames from a sequence are combined into one frame to show the change in the position of the LED throughout the frames.

The motion blur model is then applied to these images to create the final simulated image. Note that, in reality, the vehicle speed cannot stay exactly the same, as the vehicle moves. Therefore, the speed of the vehicle is assumed to change randomly within a small range in every frame. In this paper, for each series of images, the next image is simulated with vehicle speed assumed to change by 0.1% of the previous speed.

To demonstrate the result of applying the random walk and motion blur model, the simulation shown in Figure 9 is conducted with a very high degree of random change in terms of LED position and vehicle position. As a result, the LED images are greatly changed in position and blurred in some frames, as illustrated in Figure 9b.

#### 4.1.4. LED Detection and Result Evaluation

**Figure 10:**
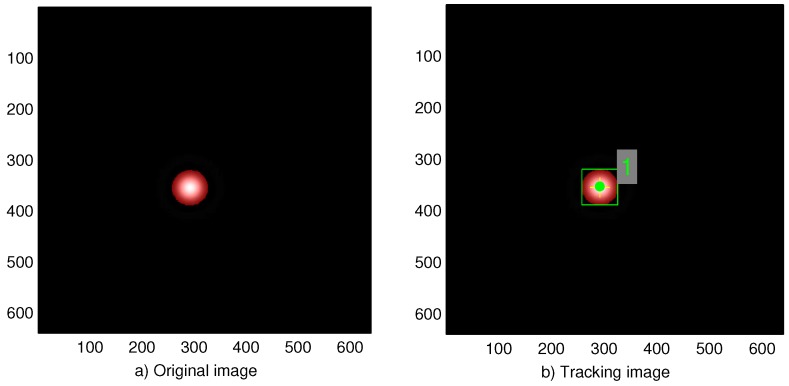
Tracking results on the image.

The case in which the position of the LED is accurately found is described in Figure 10. If the position of the LED cannot be found or is inaccurately found, this frame is considered as missing since the information embedded in the LED light cannot be extracted. Then, the rate of correct detection of the LED is calculated to evaluate the performance of the algorithm.

### 4.2. Simulation Results

**Figure 11:**
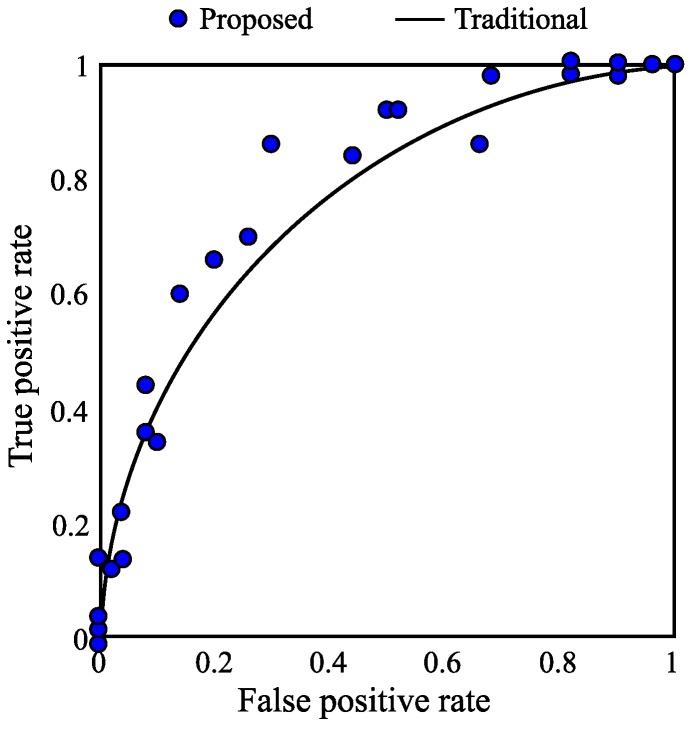
ROC of the algorithm.

A receive operating character (ROC) is plotted in Figure 11 to show the performance of the proposed tracking algorithm. The proposed algorithm was applied to a series of 100 images, and the true-positive and false-positive ratios were calculated and are represented as a dot in the figure. The curve is the best fit curve of the results of the conventional method. Since most of the results of the proposed algorithm have a higher true positive rate than the traditional algorithm, it can be confirmed that the proposed algorithm outperforms the traditional detection method.

**Figure 12:**
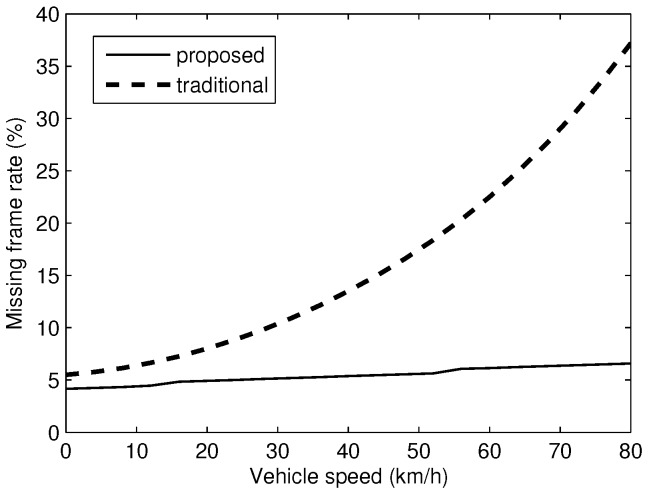
Tracking error rate over 100 images.

The missing frame rate of the proposed algorithm is shown in Figure 12. It can be seen that the missing frame rate just slightly increases when the vehicle speed increases. This is because the proposed algorithm relies on multiple information to determine the position of the LED. When the LED is blurred due to the vehicle movement, the optical flow of LED still can be constructed. By taking the optical flow of LED and the statistical information in the history into account, the calculated probability of the right LED pixel still has a higher value than that of background pixels. Therefore, the LED can be tracked successfully even though it is blurred. The traditional LED detection method relies only on pixel intensity to determine the LED position. However, when the LED image is blurred due to vehicle motion, the intensity of pixels belonging to the LED and the one belonging to the background around the LED are close in range, which leads to errors in tracking the LED. Therefore, with the traditional method, there is a noticeable increase in the missing frame when the speed increases.

## 5. Conclusions

LED detection is one of the most important and challenging steps for visible light communication using an image sensor, especially in vehicular communication systems. In such applications, high-speed cameras can be used to obtain hundreds to thousands of images of a vehicle every second, and thus the detection algorithm must be fast and accurate. Traditional detection methods that rely on pixel intensity usually find it difficult to detect the LED when the vehicle moves at a high speed since the images of the LEDs become blurred in such cases. This paper proposed a Bayesian tracking algorithm that relies on multiple type of information from the image, including optical flow and pixel intensity. Since multiple types of information, especially statistical information from past frames, are used, the LED can be detected even when the images are blurred due to a high vehicle speed. The simulated results show that the proposed algorithm is robust in tracking vehicles, even when they are moving at high speeds.
